# Adsorption of Preformed Microgel–Enzyme Complexes as a Novel Strategy toward Engineering Microgel-Based Enzymatic Biosensors

**DOI:** 10.3390/mi14081629

**Published:** 2023-08-18

**Authors:** Larisa V. Sigolaeva, Anna A. Shalybkova, Timur Z. Sharifullin, Dmitry V. Pergushov

**Affiliations:** Department of Chemistry, M.V. Lomonosov Moscow State University, Leninskie Gory 1/3, 119991 Moscow, Russia; annyshal@mail.ru (A.A.S.); timsha.359@mail.ru (T.Z.S.)

**Keywords:** microgel, enzyme, electrostatic complexation, adsorption, biosensor, triggered release, poly(*N*-isopropylacrylamide-*co*-*N*-(3-dimethylaminopropyl)methacrylamide), glucose oxidase

## Abstract

A novel approach to surface modification, which consists of the adsorption of microgel–enzyme complexes preformed in solution, is highlighted. Accordingly, the microgel–enzyme complexes were formed due to the electrostatic interaction of the oppositely charged interacting components, that is, a cationic poly(*N*-isopropylacrylamide)-based microgel and glucose oxidase taken as a model enzyme. The spontaneous adsorption of the prepared microgel–enzyme complexes, examined by means of quartz crystal microbalance with dissipation monitoring and atomic force microscopy, was observed, resulting in the formation of well-adhered microgel–enzyme coatings. Further, the preformed microgel–enzyme complexes were adsorbed onto the modified graphite-based screen-printed electrodes, and their enzymatic responses were determined by means of amperometry, demonstrating a remarkable analytical performance toward the quantification of β-D-glucose in terms of high sensitivity (0.0162 A × M^−1^ × cm^−2^), a low limit of detection (1 μM), and an expanded linear range (1–2000 μM). The fabricated microgel–enzyme biosensor constructs were found to be very stable against manifold-repeated measurements. Finally, the pH- or salt-induced release of glucose oxidase from the adsorbed preformed microgel–enzyme complexes was demonstrated. The findings obtained for the microgel–enzyme coatings prepared via adsorption of the preformed microgel–enzyme complexes were compared to those found for the microgel–enzyme coatings fabricated via a previously exploited two-stage sequential adsorption, which includes the adsorption of the microgel first, followed by the electrostatic binding of glucose oxidase by the adsorbed microgel.

## 1. Introduction

Immobilization of enzymes is one of the most important challenges of contemporary biotechnology [1,2,3,4,5]. In particular, it can be used to engineer various biosensor systems, wherein the overall biosensors’ performance is dictated by how well the enzymes are immobilized on transducers [6]. In this context, the development of approaches for the efficient and nondestructive fixation of enzymes on sensor surfaces, which, first and utmost, determines the sensitivity and operational stability of a biosensor system, is very important and in demand [7].

In many cases, gentle immobilization of enzymes on surfaces can be achieved with polymers, wherein a polymer is exploited for surface modification and a formed polymer coating can act as a “glue” (or a “host”) keeping an enzyme on the sensor surface [8]. Among various polymers, which can be advantageously applied for this purpose, are stimuli-responsive ones [9,10,11]. They can quickly and reversibly change properties with the variation in the surrounding conditions (e.g., temperature, pH, and solvent composition), which makes them very adaptive to their environment [12], including interfaces of different natures. In this context, stimuli-responsive copolymer microgels exhibiting pH and temperature dual sensitivity [13,14,15,16,17,18,19] are of considerable interest, as they are capacious containers for various payloads, including biomolecules, and, in particular, can host large amounts of enzymes, providing a favorable (highly hydrated) microenvironment.

In our previous publications [20,21,22,23,24,25], beneficial application of the pH- and temperature-sensitive copolymer microgels based on poly(*N*-isopropylacrylamide) (PNIPAM) for the efficient modification of gold and graphite (or graphite-based) surfaces and further capacious electrostatic immobilization of a number of enzymes (including multisubunit ones) was demonstrated. In those cases, the electrostatic binding of enzymes by the microgels, which resulted in the build-up of microgel–enzyme coatings, was performed at the interface when the microgels were in the adsorbed state. In this work, we propose a yet unexplored strategy to fabricate microgel–enzyme coatings, which consists of the formation of microgel–enzyme complexes in solution, followed by their adsorption onto surfaces of interest. Some possible benefits of the prepared microgel–enzyme coatings as biosensor systems are considered herein as well.

## 2. Materials and Methods

### 2.1. Materials

Glucose oxidase (GO), from *Aspergillus niger*, E.C. 1.1.3.4, activity 168,100 U/g solid was purchased from Sigma-Aldrich (Steinheim, Germany), and β-D-glucose was received from ICN Biomedicals, Inc. (Aurora, OH, USA). Tris(hydroxymethyl)aminomethane (TRIS) and its hydrochloride (TRIS-HCl) were obtained from Sigma-Aldrich (Steinheim, Germany). The sample of the poly(*N*-isopropylacrylamide-*co*-*N*-(3-dimethylaminopropyl)methacrylamide) (P(NIPAM-*co*-DMAPMA)) microgel (MS-VO-01) was synthesized by precipitation copolymerization and purified according to the procedures reported by Mergel et al. [26]. All other chemicals were of analytical grade and used without further purification. Deionized water (18.2 MΩ × cm) purified with a Milli-Q purification system from Millipore (Burlington, MA, USA) was used as a solvent for the preparation of all solutions.

### 2.2. Preparation of Microgel–Enzyme Complexes

A 10 g/L stock aqueous dispersion of the P(NIPAM-*co*-DMAPMA) microgel was prepared by a direct dissolution of the lyophilized microgel sample in water by vigorous stirring for approximately a week. An aliquot of the stock dispersion was transferred into a glass vial and diluted with 10 mM TRIS/TRIS-HCl of pH 7. The final pH of the working microgel dispersion was carefully adjusted to pH 7. A calculated volume of 1 × 10^−4^ M solution of GO in 10 mM TRIS/TRIS-HCl of pH 7 was portion-wise added to the aqueous dispersion of the P(NIPAM-*co*-DMAPMA) microgel under vigorous stirring in such a manner that the final volume of the mixture was 3 mL, the final concentration of the microgel was 1 g/L, and the ratio of the molar concentrations of GO globules to the DMAPAM moieties of the P(NIPAM-*co*-DMAPMA) microgel was set to the desired value ([GO]:[DMAPMA] = 1:80 or 1:40). The resultant mixture was stirred for 1 h before any further experiments. The prepared microgel–enzyme complexes were refrigerated at +4 °C and used within several weeks.

### 2.3. Potentiometric Titration

Potentiometric titration of an aqueous dispersion (2 mL) of the P(NIPAM-*co*-DMAPMA) microgel was performed using a digital pH meter Ohaus Starter 5000 (Ohaus Corp., Parsippany, NJ, USA) equipped with a glass pH electrode in a glass titration cell. The initial concentration of the microgel in the dispersion was 6 g/L. The initial pH value of the P(NIPAM-*co*-DMAPMA) microgel dispersion was approximately 9. The titration was carried out manually by adding portion-wise 10 mM HCl (10 μL each) to the microgel dispersion at a constant temperature of 25 °C and under vigorous stirring. Each pH value was recorded upon reaching a constant value after the addition of the successive portion of the titrant. The degree of protonation (α) of the P(NIPAM-*co*-DMAPMA) microgel was calculated from the dependence of the pH on the volume of the added titrant. The starting and the inflection points on the obtained potentiometric titration curve were taken as α = 0 and α = 1, respectively.

### 2.4. Dynamic Light Scattering and Laser Microelectrophoresis

The hydrodynamic sizes (diameters) and electrophoretic mobilities were measured at a constant temperature of 25 °C on a NanoZS Zetasizer (Malvern Instr. Ltd., Malvern, UK) via the M3-PALS technique with a HeNe laser operating at λ = 633 nm in disposable capillary cells (Malvern, DTS1070C). The detection angle was 13° and 173° for the measurements of the electrophoretic mobilities and hydrodynamic sizes, respectively. The average of 6–9 measurements was presented as the mean ± SD.

### 2.5. Fabrication of Electrochemical Microgel–Enzyme Constructs

The screen-printed electrodes (SPEs) were fabricated on poly(vinyl chloride) substrates of 0.2 mm thickness using conductive carbon graphite paste C2050517D1 from Gwent Group Co. (Pontypool, UK) screen-printed by a semi-automated Winon machine model WSC-160B (Winon Industrial Co., Ltd., Hong Kong, China) with a 200 mesh screen stencil. Each SPE consisted of a round-shaped working area (2.5 mm diameter), a conductive track (30 mm × 1.5 mm), and a square extremity (3 mm × 7 mm) for electrical contact. The SPEs were pre-modified with a thin peroxide-sensitive layer of manganese dioxide nanoparticles according to a procedure described elsewhere [23,27]. The SPE/MnO_2_ electrodes were stored dry at ambient temperature until further use. The microgel–enzyme complexes were adsorbed onto the SPE/MnO_2_ electrodes from a drop of 10 μL that was deposited on an active area of the SPE for 1 h (adsorption conditions: 10 mM TRIS/TRIS-HCl of pH 7 at 25 °C). After that time, the SPE/MnO_2_/Microgel-GO constructs were gently rinsed with Milli-Q water and shortly blown by a stream of air. For comparison, SPE/MnO_2_/Microgel/GO constructs were prepared by dipping the SPE/MnO_2_ electrodes into a 1 g/L aqueous dispersion of the microgel (adsorption conditions: 10 mM TRIS of pH 9.5 at 25 °C or 10 mM TRIS/TRIS-HCl of pH 7 at 25 °C) for 1 h, followed by gentle rinsing with Milli-Q water and short blowing by a stream of air. Afterwards, the GO was adsorbed in a similar way from 5 × 10^−5^ M solution (adsorption conditions: 10 mM TRIS/TRIS-HCl of pH 7 at 25 °C) for 10 min, followed by gentle rinsing with Milli-Q water and shortly drying with a stream of air. To prevent the loss of enzymatic activity, both the SPE/MnO_2_/Microgel-GO and the SPE/MnO_2_/Microgel/GO constructs were stored at +4 °C until further use.

### 2.6. Electrochemical Assay

Electrochemical experiments were performed at ambient temperature in a one-compartment electrochemical cell with stirring (volume of 1 mL), using a two-electrode configuration. The SPE/MnO_2_/Microgel-GO or SPE/MnO_2_/Microgel/GO constructs with an active surface area of 0.049 cm^2^ were used as a working electrode, and Ag/AgCl (with length of 1 cm, diameter of 3 mm, and surface area of 1.03 cm^2^) was a reference electrode. A potentiostat IPC Compact (Kronas Ltd., Moscow, Russia) applied for amperometric measurements was interfaced with a PC, and the electrochemical parameters were controlled by potentiostat software version IPC2000. The amperometric responses were assayed in a 50 mM HEPES/30 mM KCl buffer (pH 7.5) by recording the current arising after the addition of a solution of β-D-glucose with a standard concentration (0.5 mM in the cell). The oxidative current was generated in response to the addition of a substrate (β-D-glucose) solution at an applied potential of +450 mV vs. Ag/AgCl [27]. Each electrochemical response was determined as a value of the steady-state baseline current change (the difference between the average value of the steady-state baseline current before and after addition of β-D-glucose). The results of the amperometric measurements were represented as the mean ± SD calculated in each case for 4–5 measurements obtained for at least three similarly fabricated SPE/MnO_2_/Microgel-GO or SPE/MnO_2_/Microgel/GO constructs. In the case of pH- or salt-induced release experiments, the amperometric responses of the microgel–enzyme biosensor coatings were analyzed before and after their treatment. The treatment was carried out via incubation of the constructs for 3 or 10 min under intense stirring in 10 mM TRIS of pH 9.5 or in 150 mM or 500 mM solution of sodium chloride in water. Each release experiment was carried out in triplicate, and the obtained data are presented as the mean ± SD (n = 3). The release efficiency was calculated as 100% × (I_0_ − I_r_)/I_0_, where I_0_ is the initial enzymatic response, and I_r_ is the residual enzymatic response after the treatment.

### 2.7. Quartz Crystal Microbalance with Dissipation Monitoring (QCM-D)

The adsorption of the preformed microgel–enzyme complex, the microgel, or GO was followed in situ by means of QCM-D Q-Sense E1 system (Bioline Scientific, Gothenburg, Sweden) equipped with a Q-Sense flow-through cell. The sensor crystals (Q-Sense) were gold-coated AT-cut quartz with gold-plated polished electrodes. The quartz crystals were excited at their fundamental frequency (*F*_0_ ≈ 5 MHz) as well as at the third, fifth, seventh, ninth, and eleventh overtones, corresponding to 15, 25, 35, 45, and 55 MHz, respectively. Before use, the crystals were cleaned according to the Q-Sense cleaning protocol described elsewhere [23]. Each QCM-D experiment was carried out at 25 °C in the flow-through cell and was started from the baseline recording for the corresponding solvent (10 mM TRIS/TRIS HCl of pH 7 or 10 mM TRIS of pH 9.5). Then, the preformed microgel–enzyme complex, the microgel, or GO was adsorbed at the specified pH value (pH 7 or 9.5), followed by a washing step with the same solvent (10 mM TRIS/TRIS HCl of pH 7 or 10 mM TRIS of pH 9.5) to remove any loosely attached material. During each stage, frequency *F* and dissipation *D* shifts were continuously recorded as a function of time. Each QCM-D experiment was carried out at least twice.

### 2.8. Atomic Force Microscopy (AFM)

Freshly cleaved highly oriented pyrolytic graphite (HOPG) was used for visualization of the microgel and microgel–enzyme coatings by means of AFM. The P(NIPAM-*co*-DMAPMA) microgel or its complexes with GO were adsorbed onto HOPG (slices with the size of 5 mm × 10 mm) at ambient temperature by covering the substrate with a 20–40 μL drop of the corresponding sample, followed by adsorption for 1 h. After that time, the substrate was gently rinsed with Milli-Q water and shortly blown by a stream of air. AFM images were taken with a commercial atomic force microscope Asylum MFP-3D-SA (Asylum Research, Santa Barbara, CA, USA) operating in a semicontact mode in air. The cantilevers (fpN 10S (Super), F.V. Lukin State Research Institute for Problems in Physics, Zelenograd, Russia) with a tip curvature radius ≤10 nm, a tip cone angle ≤22°, and a resonance frequency of 190–325 kHz were used. The obtained results were presented as typical images chosen on the basis of at least five 5 μm × 5 μm uniform-sized images obtained from different places of each AFM sample. They were initially flattened using the MFP3D software version 120804 + 2702 (Asylum Research, Santa Barbara, CA, USA) run in Igor Pro 6.36 environment and then further analyzed with the Gwiddion 2.62 program. Lateral diameters and heights of the adsorbed microgel or microgel–enzyme complex particles were calculated for the separated ones.

## 3. Results and Discussion

**Figure 1:**
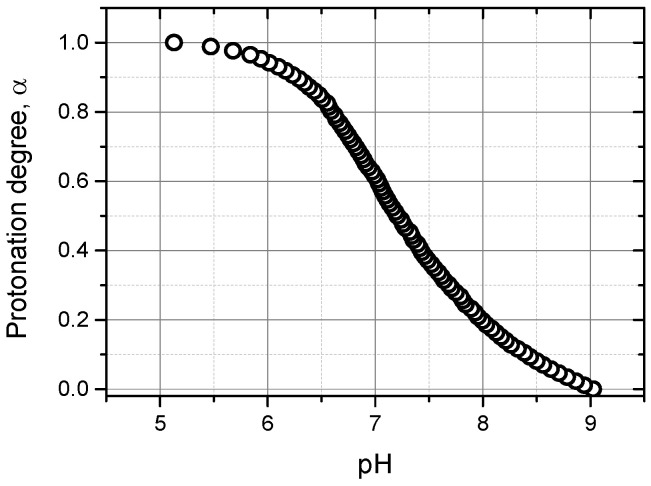
Degree of protonation (α) of the P(NIPAM-*co*-DMAPMA) microgel as a function of the pH of its aqueous dispersion. Titration was carried out via portion-wise additions of 10 mM HCl to an aqueous dispersion of the P(NIPAM-*co*-DMAPMA) microgel.

In this work, we used a PNIPAM-based cationic microgel, P(NIPAM-*co*-DMAPMA), that was synthesized as described elsewhere [26] by precipitation copolymerization of *N*-isopropylacrylamide (NIPAM) and a cationic comonomer dimethylaminopropyl methacrylamide (DMAPMA), whose content in the reaction mixture was set to 10% (mol.), in the presence of a crosslinking agent *N*,*N*’-methylenebisacrylamide. Being dissolved in aqueous media, this microgel forms opalescent dispersions that are stable on the colloidal level. Potentiometric titration of the P(NIPAM-*co*-DMAPMA) microgel was performed to quantify the amount of the DMAPMA comonomer, which was included in the microgel upon its preparation, and to reveal the pH window corresponding to the reversible protonation–deprotonation of the DMAPMA units (Figure 1). The actual content of the DMAPMA units in the microgel was found to be about 9% (mol.), which is close to the amount taken for the synthesis. As seen, the full protonation of the P(NIPAM-*co*-DMAPMA) microgel was observed at pH ≤ 5.2, while all the DMAPMA units in the microgel were deprotonated at pH ≥ 9, being in good accordance with the former findings reported elsewhere [20,23].

**Figure 2:**
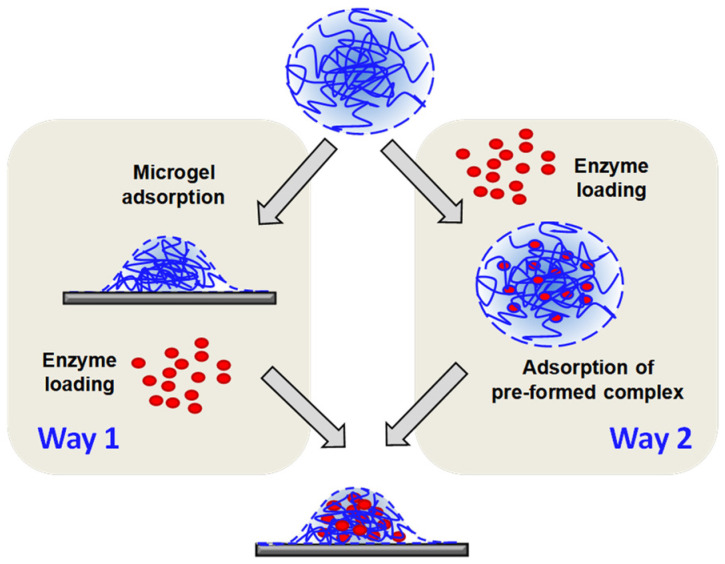
Two strategies for preparation of microgel–enzyme coatings: Way 1 (**left**) is two-stage sequential adsorption, wherein a microgel is first deposited onto surface, followed by loading of the adsorbed microgel with an enzyme; Way 2 (**right**) includes first the formation of a microgel–enzyme complex in solution, followed by its adsorption onto the surface.

Previously, we exploited **two-stage sequential adsorption** (Figure 2, Way 1) for engineering the microgel–enzyme coatings. Specifically, the P(NIPAM-*co*-DMAPMA) microgel was first deposited onto a hydrophobic (or moderately hydrophobic) surface (like graphite or gold) under the conditions when the microgel was considerably hydrophobized, that is, at high pH values and (optionally) an elevated temperature. Then, an enzyme was loaded (via electrostatic immobilization) into the P(NIPAM-*co*-DMAPMA) microgel at “mild” conditions (typically, ambient temperature and neutral pH), provided that the microgel and the enzyme were oppositely charged. As described in our former publications [20,21,22,23,24,25], maintaining the right (“best”) conditions at any stage of fabrication of the microgel–enzyme coatings is very important to provide a large amount of the enzyme at the surface, which is safely immobilized into the adsorbed microgels via the electrostatic binding.

Another way to fabricate the microgel–enzyme coatings includes at first complex formation between the microgel and the enzyme in solution at the pH values, wherein they are oppositely charged and can form complexes stabilized via the electrostatic interaction, followed by **adsorption of the preformed complex** (Figure 2, Way 2). In this work, we address this yet unexplored strategy for engineering the microgel–enzyme coatings.

The GO used in this work as a model enzyme is a dimer consisting of two subunits, which has a low isoelectric point (pI = 4.2) and therefore a net negative charge at pH values exceeding pI, the negative charges being localized on the enzyme surface and dimer interface [28]. At pH 7, the positively charged P(NIPAM-*co*-DMAPMA) microgel (Figure 1) can bind negatively charged GO, thereby forming the microgel–enzyme complex. Hence, two complexes were prepared upon direct mixing of the solutions of the components at pH 7 and two different [GO]:[DMAPMA] ratios of 1:80 and 1:40, where [GO] is the molar concentration of enzyme globules, while [DMAPMA] is the molar concentration of the cationic comonomer units. As one can see from Table 1, the formation of the microgel–enzyme complexes at [GO]:[DMAPMA] = 1:40 clearly manifested itself by considerable changes in the hydrodynamic size (D_h_) of the P(NIPAM-*co*-DMAPMA) microgel and its electrophoretic mobility (μ_E_) (or ζ-potential) in its mixtures with GO. The increased hydrodynamic size and the lowered electrophoretic mobility of the microgel–enzyme complex at [GO]:[DMAPMA] of 1:40 point to the formation of aggregated complex structures when the P(NIPAM-*co*-DMAPMA) microgel’s charge was sufficiently compensated by the enzyme’s charge upon binding of the GO. At the same time, no significant difference between the pristine microgel and the microgel–enzyme complex at [GO]:[DMAPMA] of 1:80 strongly suggests that the microgel’s surface charge was not yet compensated by the charge of the GO globules, which apparently tend to be predominantly localized in the inner part of each microgel.

Quartz crystal microbalance with dissipation monitoring (QCM-D) was exploited to reveal the main features of formation of the microgel–enzyme coatings prepared according to the abovementioned two different strategies depicted in Figure 2. As demonstrated in our former publications [20,22,23], this technique allows one to follow the online (in real-time mode) deposition of matter onto gold surface of a QCM-D sensor via monitoring the changes in its resonance frequency *F* and dissipation *D* as a function of time in a flow-through cell.

**Figure 3:**
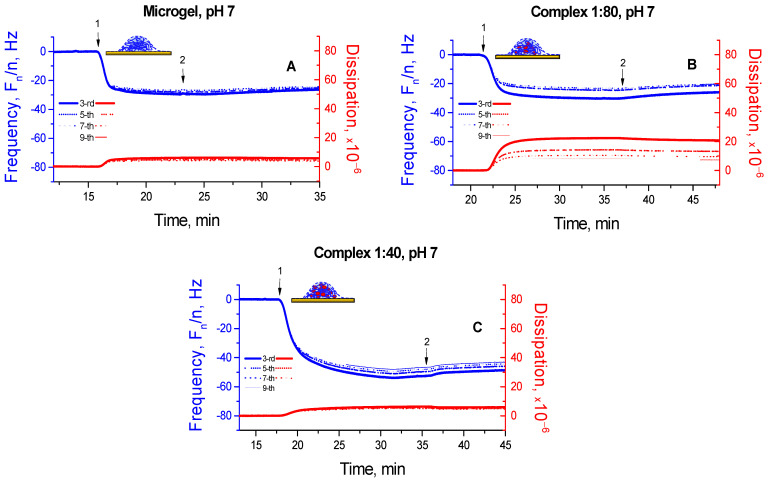
Normalized frequency *F_n_/n* (blue lines) and dissipation *D* (red lines) shifts for the gold-coated quartz crystal upon the adsorption at pH 7 of the P(NIPAM-*co*-DMAPMA) microgel (**A**); the preformed microgel–enzyme complex at [GO]:[DMAPMA] = 1:80 (**B**); and the preformed microgel–enzyme complex at [GO]:[DMAPMA] = 1:40 (**C**). 1—adsorption of the pristine microgel (**A**) or the microgel–enzyme complex (**B**,**C**) in 10 mM TRIS/TRIS-HCl of pH 7; 2—washing with 10 mM TRIS/TRIS-HCl of pH 7. The experiments were carried out at 25 °C.

Adsorption of the P(NIPAM-*co*-DMAPMA) microgel at pH 7 induced some negative shifts in *F*-values and simultaneous positive shifts in *D*-values, both with certain spreading of overtones (Figure 3A). This indicates that the formed microgel coating was viscoelastic, although the changes in the *F*- and *D*-values appear to be rather weak. This result is in line with our former reports on the pH-dependent interaction of ionic (cationic or anionic) microgels with solid surfaces like gold or graphite (graphite-based ones) [23,29], showing a weak adsorption of such microgels in their charged state. The adsorption at the same pH of the microgel–enzyme complex preformed at [GO]:[DMAPMA] = 1:80 also induces changes in the *F*- and especially *D*-values, accompanied by a stronger spreading of overtones (Figure 3B). Even more pronounced shifts of *F*-values were observed upon adsorption at pH 7 of the microgel–enzyme complex preformed at [GO]:[DMAPMA] = 1:40 (Figure 3C). However, the changes in the *D*-values were weaker in this case than those observed for the microgel–enzyme complex preformed at [GO]:[DMAPMA] = 1:80, and the spreading of overtones was clearly suppressed, both strongly suggesting that the film composed of the preformed microgel–enzyme complex with the higher content of GO was more rigid. Remarkably, washing (ca. 5–10 min) with the solvent (10 mM TRIS/TRIS-HCl of pH 7) after adsorption was accompanied by negligible changes in the *F*- and *D*-values, thereby indicating that neither the adsorbed pristine microgels nor the adsorbed microgel–enzyme complexes left the surface. Hence, all these findings evidence that the preformed microgel–enzyme complexes can spontaneously adsorb onto surfaces like gold and form well-adhered microgel–enzyme coatings that are stable against flush-off treatment.

**Figure 4:**
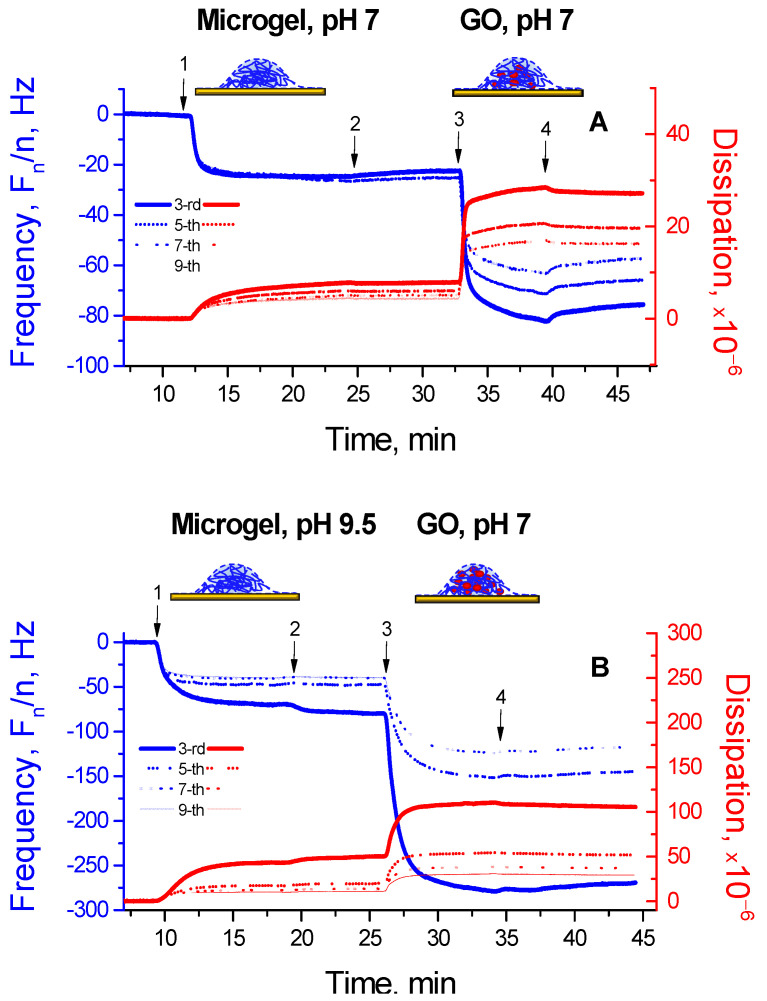
Normalized frequency *F_n_/n* (blue lines) and dissipation *D* (red lines) shifts for a gold-coated quartz crystal upon the sequentially adsorbed P(NIPAM-*co*-DMAPMA) microgel at pH 7 and then GO at pH 7 (**A**) and the sequentially adsorbed P(NIPAM-*co*-DMAPMA) microgel at pH 9.5 and then GO at pH 7 (**B**). 1—adsorption of the pristine microgel in 10 mM TRIS/TRIS-HCl of pH 7 (**A**) or 10 mM TRIS of pH 9.5 (**B**); 2—washing with 10 mM TRIS/TRIS-HCl of pH 7 (**A**) or 10 mM TRIS of pH 9.5 (**B**); 3—adsorption of GO in 10 mM TRIS/TRIS-HCl of pH 7; 4—washing with 10 mM TRIS/TRIS-HCl of pH 7. The experiments were carried out at 25 °C.

To demonstrate differences in the microgel–enzyme coatings prepared via different strategies, similar QCM-D experiments were carried out for two-stage sequential adsorption according to Way 1 (Figure 2, left). Here, the P(NIPAM-*co*-DMAPMA) microgel was adsorbed first at the specified pH value, that is, pH 7 (Figure 4A) or pH 9.5 (Figure 4B), followed by a washing step with the solvent (10 mM TRIS/TRIS-HCl of pH 7 or 10 mM TRIS of pH 9.5, respectively) to remove any loosely attached material. Then, the formed microgel coating was brought into contact with a solution of GO at pH 7, and the experiments were finalized by a washing step with 10 mM TRIS/TRIS-HCl of pH 7 (Figure 4A,B).

It is worth noting that the observed changes in the *F*- and *D*-values were much more pronounced in the case of adsorption of the P(NIPAM-*co*-DMAPMA) microgel at pH 9.5 than at pH 7. This fact indicates that the amount of the adsorbed microgel was strongly opposite in trend to the microgel’s charge. The latter was determined by the pH value at which the adsorption of the P(NIPAM-*co*-DMAPMA) microgel was performed. The best surface modification was achieved when the microgel was adsorbed onto the gold surface of the QCM-sensor at pH 9.5, that is, when it was in the uncharged (and consequently hydrophobized) state, which is in line with our former findings [23,29]. The further evolution of the *F*- and *D*-values upon the electrostatic binding of GO at pH 7 showed a pronounced decrease in the *F*-values with a concomitant increase in the *D*-values when the enzyme was loaded into the adsorbed P(NIPAM-*co*-DMAPMA) microgels, with the changes in the *F*- and *D*-values being much stronger when the microgel was initially adsorbed at the higher pH (pH 9.5). Hence, the loading of the microgel coating with GO appears to directly correlate with the efficiency of the surface modification by the P(NIPAM-*co*-DMAPMA) microgel on the previous stage.

Generally, one can note a more rigid character of the microgel–enzyme coatings, which are fabricated according to Way 2, that is, adsorption of the preformed microgel–enzyme complex (Figure 2, right), compared with highly viscoelastic microgel–enzyme coatings, which are prepared according to Way 1, that is, two stage sequential adsorption first of the microgel and then the enzyme (Figure 2, left), as indicated by the lower *D*-values with rather weak spreading of overtones (cf. Figure 3 and Figure 4).

**Figure 5:**
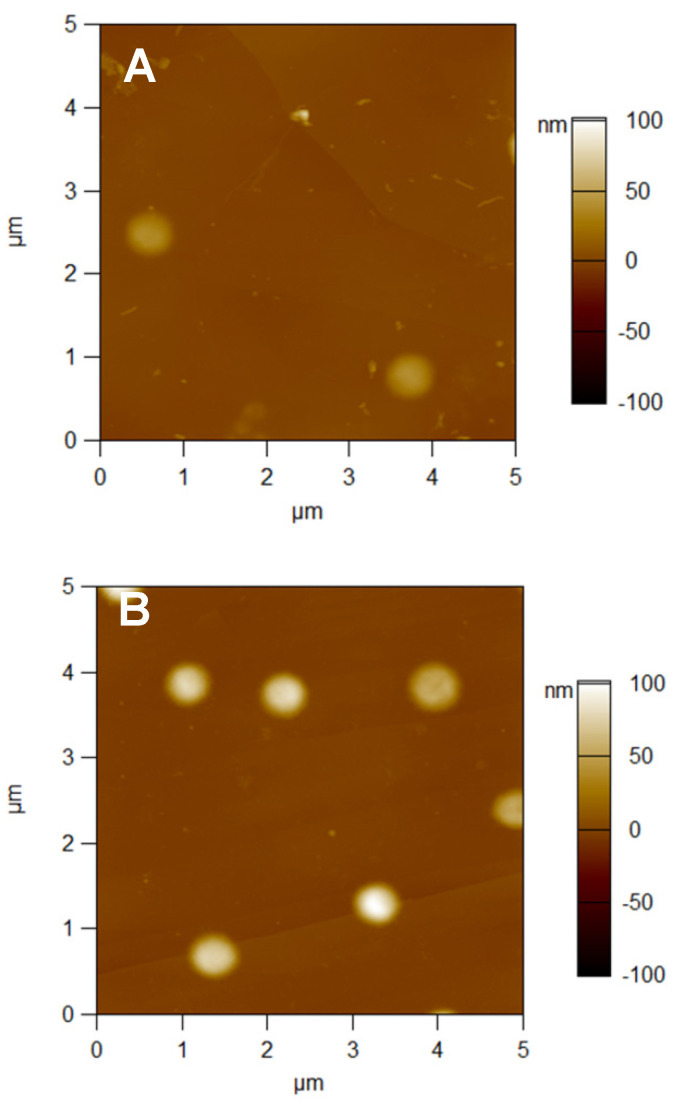

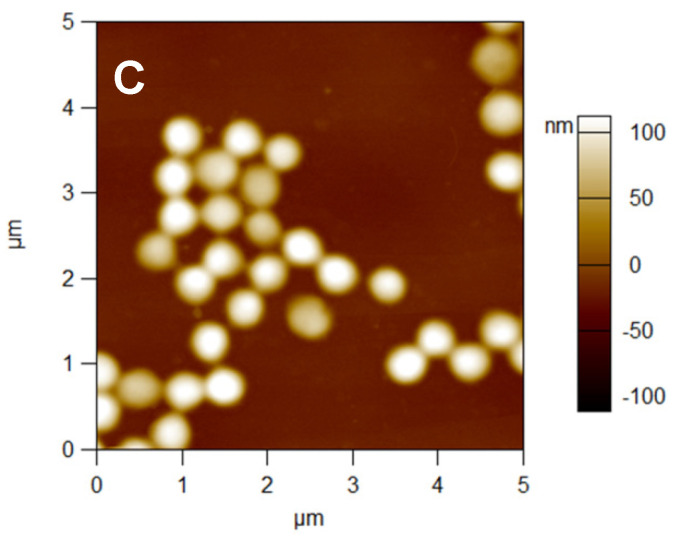
The 5 μm × 5 μm AFM height images of the P(NIPAM-*co*-DMAPMA) microgel (**A**), the microgel–enzyme complexes preformed at [GO]:[DMAPMA] = 1:80 (**B**), and at [GO]:[DMAPMA] = 1:40 (**C**), which were adsorbed onto HOPG from solutions in 10 mM TRIS of pH 7, 25 °C for 1 h. The images were taken in a dry state.

The adsorbed P(NIPAM-*co*-DMAPMA) microgel and its complexes with GO were visualized by means of atomic force microscopy (AFM) on HOPG. Individual round-shaped objects, which are present in all images (Figure 5), can obviously be attributed to microgel particles. Only a few of them were observed on the surface when the pristine P(NIPAM-*co*-DMAPMA) microgel was adsorbed at pH 7 (Figure 5A). This finding, which indicates a poor adsorption of the charged microgels onto sufficiently hydrophobic solid surfaces, is in line with our results obtained by means of the QCM-D (Figure 3A) as well as with our former publications [23,29]. At the same time, the number of the round-shaped objects clearly increased when the microgel–enzyme complexes were adsorbed (Figure 5B,C), being pronouncedly higher at the ratio [GO]:[DMAPMA] of 1:40. This strongly suggests that the increased loading of the P(NIPAM-*co*-DMAPMA) microgel with GO favored the adsorption of the microgel–enzyme complexes. One can also see that particles of the microgel–enzyme complex preformed at the ratio [GO]:[DMAPMA] of 1:80 were well-separated (Figure 5B), while those of the microgel–enzyme complex preformed at the ratio [GO]:[DMAPMA] of 1:40 were clusters of closely packed objects (Figure 5C). The latter appears to come from a presence of the aggregated structures in dispersions of the microgel–enzyme complex (Table 1).

The evaluated numbers of the round-shaped objects per scan (the adsorbed P(NIPAM-*co*-DMAPMA) microgel and its complexes with GO) are summarized in Table 2 along with their size/shape characteristics. Indeed, the pristine microgel (Figure 5A) appeared in the images as more flattened (thin) objects having a height of about 44 nm, while the microgel–enzyme complexes (Figure 5B,C) appeared as less flattened (thicker) objects with heights of about 84 nm and 133 nm for [GO]:[DMAPMA] = 1:80 and 1:40, respectively. These changes in heights were accompanied by a notable decrease in the diameters of the objects.

We further examined the enzymatic activity of the GO immobilized into the P(NIPAM-*co*-DMAPMA) microgel coating via electrochemical (amperometric) measurements. For this, the preformed microgel–enzyme complexes (Figure 2, right) were adsorbed onto surface of graphite-based SPEs modified by a thin mediator layer of manganese dioxide nanoparticles. Simultaneously, a reference system was fabricated, wherein a SPE/MnO_2_ electrode was modified via two-stage sequential adsorption (Figure 2, left). The enzymatic responses of the GO were measured as the oxidative current, which is generated in response to the addition of β-D-glucose at an applied potential of +450 mV vs. a Ag/AgCl reference electrode [23] (see more details of electrochemical measurements in Figures S1 and S2 of Supplementary Materials). The obtained electrochemical responses are summarized in Table 3.

Clearly, the enzymatic responses are comparable for both systems. Here, we interpret the amperometric responses as values that are directly proportional to the amount of GO incorporated into the adsorbed P(NIPAM-*co*-DMAPMA) microgel. Within each system, they appear to correlate with the data obtained by means of the QCM-D and AFM. In the case of the microgel–enzyme coatings prepared via Way 1 (Figure 2, left), the biosensor responses were impacted by the pH condition at which the adsorption of the microgel (a “host”) takes place. In the case of the microgel–enzyme coatings fabricated via Way 2 (Figure 2, right), the biosensor responses were determined by the content of the GO (a “guest”) in the preformed microgel–enzyme complex. One can also note that the SD-value for the amperometric response of the SPE/MnO_2_/Microgel-GO constructs at [GO]:[DMAPMA] = 1:40 was higher than that found for the constructs at [GO]:[DMAPMA] = 1:80, which most probably comes from the presence of the aggregated complex structures as mentioned above.

**Figure 6:**
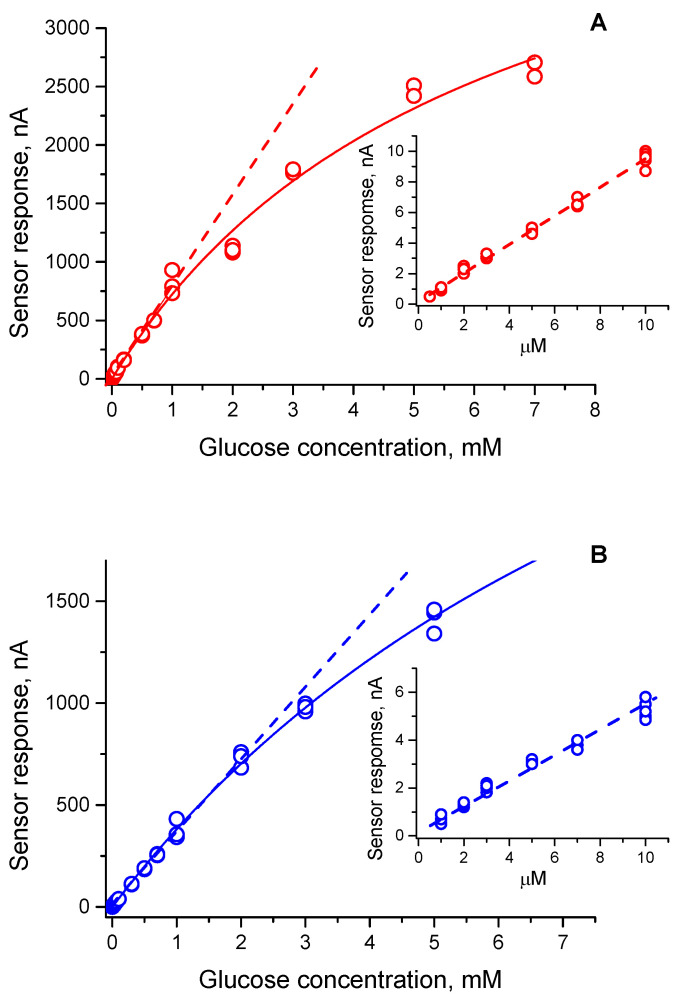
The dependence of the steady-state current on the β-D-glucose concentration for SPE/MnO_2_/Microgel (pH 9.5)/GO (**A**) and SPE/MnO_2_/Microgel-GO ([GO]/[DMAPMA] = 1:80) (**B**) constructs. Conditions: 50 mM HEPES/30 mM KCl (pH 7.5), room temperature. Linear regression: Y = (1.4 × 10^−3^ ± 3.5 × 10^−3^) + (785.7 ± 12.0) × X, R^2^ = 0.9869 (**A**), Y = (2.1 × 10^−3^ ± 1.7 × 10^−3^) + (365.6 ± 3.2) × X, R^2^ = 0.9955 (**B**), where Y represents the current in nA and X represents the β-D-glucose concentration in mM. Inserts: initial part of the calibration curves. The solid lines are drawn through the experimental points only as a guide for the eyes. The dashed lines represent the corresponding linear regressions.

The analytical characteristics toward detection of β-D-glucose were evaluated for the microgel–enzyme coatings fabricated according to Way 1 (SPE/MnO_2_/Microgel (pH 9.5)/GO) and according to Way 2 (SPE/MnO_2_/Microgel-GO at [GO]:[DMAPMA] = 1:80). The original amperometric responses measured at different substrate (β-D-glucose) concentrations are shown in Figure S2 of the Supplementary Materials. As one can see, the response time was fast. Indeed, approximately 90% of the steady-state current was already achieved within 10 s. The change in the steady-state baseline current for both systems as a function of the β-D-glucose concentration is presented in Figure 6, while Table 4 gives a detailed comparison of their analytical characteristics such as the sensitivity, linear range, and the limit of detection. It is evident that both microgel–enzyme constructs demonstrated high sensitivity toward detection of β-D-glucose, despite being different by a factor of two from each other. The obtained calibration curves show linearity in a range of three orders of magnitude (Figure 6) and the limit of detection at a signal-to-noise ratio (S/N) of 3 as low as 0.5–1 μM (Table 4). It is worth noting that the analytical performance of our microgel–enzyme biosensor constructs is remarkable, and they are among the best polymer/nanomaterials-based biosensors containing glucose oxidase as, in particular, follows from comparison with the other systems reported elsewhere (see the reviews [30,31] and references therein).

**Figure 7:**
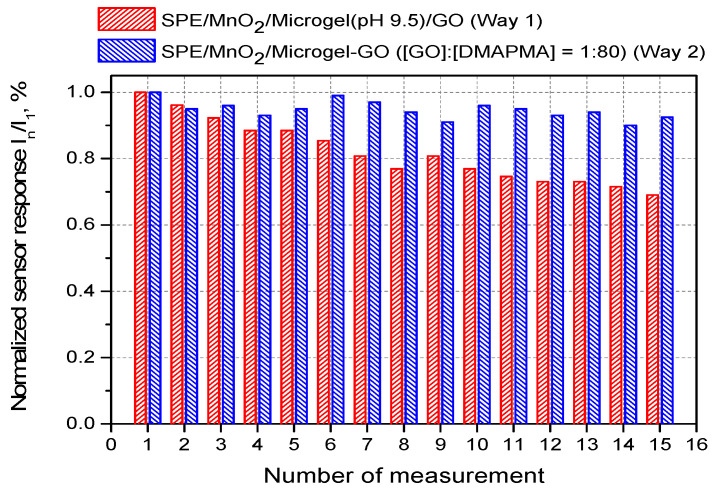
The relative enzymatic responses for the microgel–enzyme coatings fabricated via two-stage sequential adsorption first of the P(NIPAM-*co*-DMAPMA) microgel (pH 9.5) and then of GO (pH 7) (red columns) and the microgel–enzyme coatings fabricated via adsorption of the microgel–enzyme complex preformed at [GO]:[DMAPMA] = 1:80 (pH 7) (blue columns) to sequential additions of β-D-glucose in a concentration of 0.5 mM.

Further comparing both systems, we found that the microgel–enzyme coatings prepared via Way 2 (Figure 2, right) exhibited almost invariant enzymatic responses over repeated measurements, while those for the microgel–enzyme coatings fabricated via Way 1 (Figure 2, left) gradually decreased from measurement to measurement (Figure 7). The enzymatic response stability was characterized quantitatively as percentage of its change per single measurement and was calculated according to the formula: Δ = 100% × tgI/I_1_, where tgI is the slope of the dependence of the enzymatic response on the number of measurements normalized to the initial response I_1_ and is given in percent. Remarkably, the loss of the enzymatic response per single measurement was only Δ = 0.30 ± 0.11% for the SPE/MnO_2_/Microgel-GO constructs, while it was higher and reached Δ = 2.14 ± 0.18% for the SPE/MnO_2_/Microgel (pH 9.5)/GO constructs.

One can assume that the observed loss of the enzymatic activity might be caused by a gradual release of a certain (loosely bound) fraction of the GO from the adsorbed P(NIPAM-*co*-DMAPMA) microgel upon the repeated measurements. Such release can be facilitated by weakening (or even completely cancelling) the electrostatic interaction between the oppositely charged microgel and enzyme via variations in, for example, the ionic strength or the pH of the surrounding medium. In particular, salt- or pH-induced release of various payloads (both high and low molecular mass ones) was described previously elsewhere [29,32,33,34].

**Figure 8:**
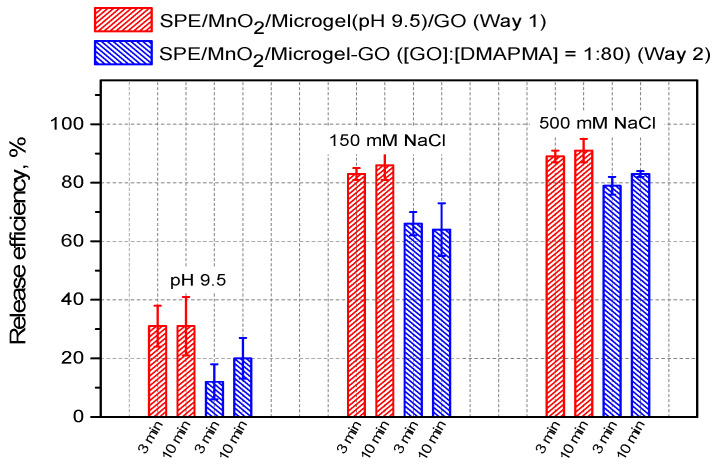
The efficiency of the release of GO from the P(NIPAM-*co*-DMAPMA) microgel measured for the microgel–enzyme coatings fabricated via a two-stage sequential adsorption first of the P(NIPAM-*co*-DMAPMA) microgel (pH 9.5) and then of the GO (pH 7) (red columns) and the microgel–enzyme coatings fabricated via adsorption of the microgel–enzyme complex preformed at [GO]:[DMAPMA] = 1:80 (pH 7) (blue columns) at different release conditions. The release efficiency was calculated as 100% × (I_0_ − I_r_)/I_0_, where I_0_ is the initial enzymatic response, and I_r_ is the residual enzymatic response after the corresponding treatment. The data are presented as the mean ± SD for three independent experiments.

In this work, we performed a comparative examination of the release of GO from the adsorbed P(NIPAM-*co*-DMAPMA) microgel for the microgel–enzyme coatings prepared via Way 2 (Figure 2, right) and Way 1 (Figure 2, left). Specifically, they were subjected to a 3 min or 10 min treatment by 10 mM TRIS with pH 9.5 (full discharging of the microgel) or by 150 mM or 500 mM NaCl in water (screening the electrostatic interaction between the microgel and the enzyme by small ions). The enzymatic responses were analyzed before and after the treatment stage, and the release efficiency was calculated as 100% × (I_0_ − I_r_)/I_0_, where I_0_ is the initial enzymatic response, and I_r_ is the residual enzymatic response after treatment. Figure 8 shows the release efficiencies for the microgel–enzyme coatings fabricated via two considered ways at different release conditions. One can see that the release of GO from the adsorbed P(NIPAM-*co*-DMAPMA) microgel was not complete regardless of the way used for fabrication of the microgel–enzyme coatings and the treatment applied. Further, the release was rather fast, as nearly no difference was observed between the 3 min and 10 min treatments, except for the treatment of the SPE/MnO_2_/Microgel-GO constructs at pH 9.5. Finally and most importantly, the microgel–enzyme coatings fabricated according to Way 2 (Figure 2, right) appeared to always lose less GO compared to those fabricated according to Way 1 (Figure 2, left) at any release conditions applied herein. These findings strongly suggest that GO is retained in the adsorbed P(NIPAM-*co*-DMAPMA) microgel more efficiently when its immobilization into the microgel is performed in a bulk solution rather than at the interface.

## 4. Conclusions

Herein, we highlight a successful approach to surface modification, which consists of the adsorption of microgel–enzyme complexes preformed in solution. We demonstrate that at pH 7 the P(NIPAM-*co*-DMAPMA) microgel binds GO (a model enzyme) due to the electrostatic interaction of the oppositely charged interacting components. Further, we show that such preformed microgel–enzyme complexes at pH 7 spontaneously adsorb onto surfaces like gold and graphite, generating the microgel–enzyme coatings. Enzymatic responses of GO in the microgel–enzyme complexes adsorbed onto the graphite-based electrodes were measured via electrochemical assay (amperometry), demonstrating remarkable analytical performance (sensitivity, the limit of detection, and the linear range) toward quantification of β-D-glucose. Moreover, notably more stable enzymatic responses against manifold repeated measurements as well as the hampered pH- or salt-induced release of the loaded enzyme from the adsorbed P(NIPAM-*co*-DMAPMA) microgel were found, compared to those observed for the microgel–enzyme coatings fabricated via a sequential two-stage adsorption procedure, which was previously exploited.

Hence, these findings might be important when considering applications of such microgel–enzyme coatings as highly sensitive electrochemical (e.g., glucose) biosensors, especially for analysis of real samples, which might inter alia contain ions as interferents. On the other hand, one should note that the microgel–enzyme coatings fabricated via two-stage sequential adsorption, which exhibit stronger depletion of GO, can be of interest for the development of (prolonged) release systems for various applications (e.g., in medicine or agriculture).

## Figures and Tables

**Table 1:** The hydrodynamic diameter (D_h_), the electrophoretic mobility (μ_E_), and the ζ-potential of the P(NIPAM-*co*-DMAPMA) microgel and its complexes with GO. Conditions: pH 7, 25 °C.

Sample	D_h_, nm	μ_E_, (μm × cm)/(V × s)	ζ-Potential, mV
Microgel	710 ± 10	+1.249 ± 0.036	+16.0 ± 0.4
Microgel–enzyme complex [GO]:[DMAPMA] = 1:80	790 ± 70	+1.287 ± 0.051	+16.4 ± 0.7
Microgel–enzyme complex [GO]:[DMAPMA] = 1:40	1370 ± 300	+0.297 ± 0.078	+3.8 ± 1.0

**Table 2:** A comparative analysis of the AFM images taken in air for the P(NIPAM-*co*-DMAPMA) microgel and its complexes with GO adsorbed onto HOPG for 1 h at pH 7, 25 °C.

Sample	Number of Objects Per Scan ^(1)^	Object Height, nm ^(2)^	Object Diameter, nm ^(2),(3)^
Microgel	2 ± 1	44 ± 7 (n = 6)	675 ± 24 (n = 6)
Microgel–enzyme complex [GO]:[DMAPMA] = 1:80	8 ± 2	84 ± 16 (n = 30)	614 ± 54 (n = 30)
Microgel–enzyme complex [GO]:[DMAPMA] = 1:40	37 ± 7	133 ± 13 (n = 35)	593 ± 33 (n = 35)

^(1)^ Mean ± SD for at least 5 scans; ^(2)^ Mean ± SD, calculated for n objects; ^(3)^ No tip convolution was taken into account.

**Table 3:** The amperometric responses (nA) for the microgel–enzyme coatings prepared via Way 1 (Figure 2, left) and Way 2 (Figure 2, right) for a substrate (β-D-glucose) concentration of 0.5 mM. The data are presented as the mean ± SD.

Way 1		Way 2	
SPE/MnO_2_/Microgel (pH 7)/GO	120 ± 30	SPE/MnO_2_/Microgel-GO [GO]:[DMAPMA] = 1:80	250 ± 65
SPE/MnO_2_/Microgel (pH 9.5)/GO	380 ± 55	SPE/MnO_2_/Microgel-GO [GO]:[DMAPMA] = 1:40	550 ± 110

**Table 4:** Comparison of the analytical characteristics of the microgel–enzyme biosensor constructs fabricated via Way 1 (Figure 2, left) and Way 2 (Figure 2, right).

Analytical Parameter	System	
	SPE/MnO_2_/Microgel (pH 9.5)/GO	SPE/MnO_2_/Microgel-GO [GO]:[DMAPMA] = 1:80
Sensitivity, A/(M × cm^2^)	0.0162	0.00746
Linear range, μM	0.5–1000	1.0–2000
Limit of detection (S/N = 3), μM	0.5	1.0

## Data Availability

The data presented in this study are available on request from the corresponding authors.

## Supplementary Materials

The following supporting information can be downloaded at: https://www.mdpi.com/article/10.3390/mi14081629/s1, Figure S1: A principal scheme of the electrochemical analysis of the enzymatic responses of GO. Figure S2: The original amperometric responses measured at different substrate (β-D-glucose) concentrations.
