# Supplementary material for: Adsorption of Preformed Microgel–Enzyme Complexes as a Novel Strategy toward Engineering Microgel-Based Enzymatic Biosensors
Source: Micromachines (Basel). 2023 Aug 18;14(8):1629. doi: 10.3390/mi14081629 (PMC10456651; doi:10.3390/mi14081629)
Supplement: Supplementary file 1 [file micromachines-14-01629-s001.zip › micromachines-2486567-supplementary.pdf]

Supplementary Materials

Adsorption of pre-formed microgel-enzyme complexes as a novel strategy toward engineering of microgel-based enzymatic biosensors

Larisa V. Sigolaeva*, Anna A. Shalybkova, Timur Z. Sharifullin, Dmitry V. Pergushov*

Department of Chemistry, M.V. Lomonosov Moscow State University,

Leninskie Gory 1/3, 119991 Moscow, Russia

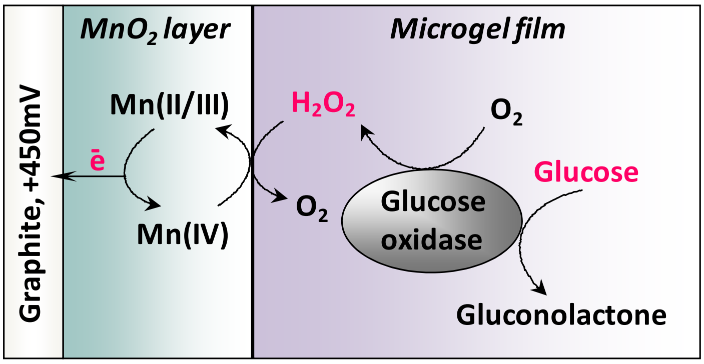

Figure S1. A principal scheme of electrochemical analysis of glucose by microgel-based biosensor.

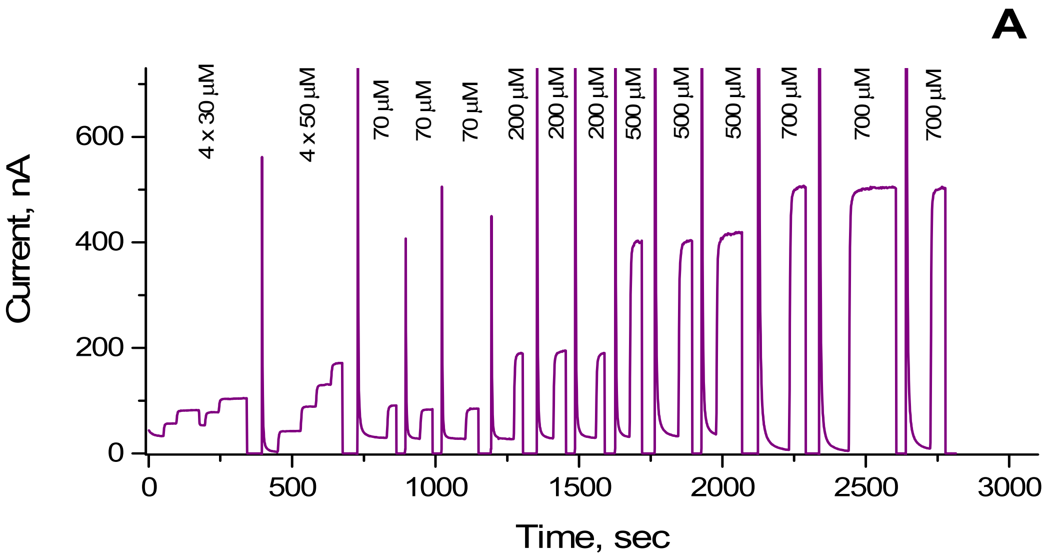

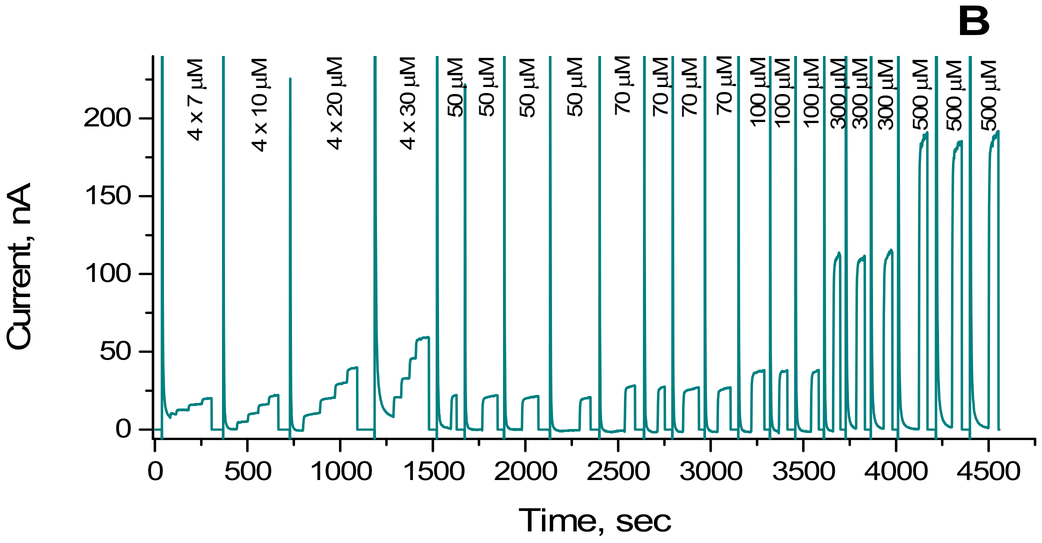

Figure S2. Responses toward addition of β-D-glucose for SPE/MnO_2/Microgel/GO (A) or SPE/MnO_2/Microgel-GO (B).
